# Lessons from a ‘low-tech’ community approach to youth mental health in Argentina: shared space, purpose and stories

**DOI:** 10.1016/j.lana.2026.101492

**Published:** 2026-05-05

**Authors:** Paola Buedo, Timothy Daly

**Affiliations:** a Institute of History and Ethics in Medicine, Department of Preclinical Medicine, TUM School of Medicine and Health, Technical University of Munich, Munich, Germany; b Bioethics Program, FLACSO Argentina, Buenos Aires, Argentina; c CNRS UMR 8011 Science Norms Democracy, Sorbonne Université, Paris, France

The increased digitalisation of global society offers both opportunities and challenges for public mental health. On the one hand, there is an evolving landscape of innovative digital mental health interventions.^1^ On the other hand, increased digitalisation of life can exacerbate exposure to sources of ill mental health due to toxic online environments, leading to dependency, increased socio-political fractures, and a deepened sense of isolation.^2^ Against the backdrop of this digital burden and structural barriers to access to adequate youth mental health care, the emergence of generative artificial intelligence (AI) has led many young people worldwide to rely on AI chatbots as their main, and unproven, source of mental health support.^3^ Moreover, in our era of anthropogenic climate change, there is a further source of value conflict relevant to youth mental health. Youths increasingly depend on digital technologies, including AI, which is associated with growing environmental concerns such as resource extraction and contribution to climate change. Yet, the majority of youths are “very or extremely worried” about climate change, leading to worsened youth mental health.^3^

A recent report from the World Health Organization (WHO) on the digital determinants of youth mental health underlines the importance of investing in ‘low-tech’ community-based interventions.^4^ Drawing on the first author's professional experience, this article describes a formal healthcare offer and an ongoing community-based approach to youth mental health promotion launched in 2013 in Bahia Blanca, Argentina: ‘You Are the Good News' (in Spanish, ‘La Buena Noticia Sos Vos’), which uses urban art to reach young people.^5^ As the group's name suggests, *La Buena Noticia* portrays young people as valuable members of society, not defined by their mental illness or risk profile. By removing diagnostic labels, the initiative enables personal growth regardless of an individual's clinical status and provides an opportunity to engage with different types of tasks, promote community engagement and diverse relationships. Coordinated by a team of physicians, psychologists, and artists, and co-led by young people, the initiative involves weekly meetings to plan and carry out public art projects, primarily mural painting. The murals are painted on the external walls of social institutions, which often bring together different groups of local people for meals and foster interaction between them. These activities, when carried out periodically in a group, promote the development of social and political skills, the adoption of responsibilities within a peer group, and the fostering of agency whilst nurturing community integration.

There are many different bottom-up approaches to youth mental health that could be appropriately tailored to given contexts. In Latin American countries, exposure to contextual determinants of poor mental health is high, and cultural aspects play a significant role in mental health development.^6^ The *La Buena Noticia* model is a locally implemented strategy that caters for cultural needs and sensitivities, relying on collective co-creation to promote mental health. Other similar community-level initiatives exist in the country, such as the ‘PILOT Project: Making a way of no way’, focusing on the relationship between music and youth brain and mental health promotion in marginalised communities in San Juan, Argentina.^7^

Mental health systems that provide this type of complementary, low-cost care are likely to achieve better health outcomes than those that focus exclusively on individual-level interventions.^8^ We consider that there are three major lessons for low-tech community-led initiatives to youth mental health promotion that other initiatives in Latin America and beyond can learn from: shared space, purpose, and stories (Table 1).

**Table 1:** The relevance of shared space, shared purpose, and shared stories for youth mental health.

Lesson	Relevance to the digital age for youth mental health	Opportunities for youth mental health promotion
Shared space	High use of AI may lead users to erroneously consider their mental health needs as detached from the physical environment. The mental health fallout of the COVID-19 pandemic demonstrated the importance of sharing physical spaces with other members of the community for mental health promotion.	While taking necessary public health precautions, initiatives should encourage shared space-sharing, as well as nature-based health promotion such as community gardening that can achieve co-benefits such as respect for the environment.
Shared purpose	Communities are defined by their shared values, a clear goal and activities, which online platforms such as social media lack. Conversely, all meetings of initiatives such as *La Buena Noticia*, whether it is to organise events, chat over a *mate*, or play the guitar together, are “always undertaken with [a] purpose”.	Art-based initiatives that organise activities based on creative expression can provide an outlet for experiencing community-level and individual meaning and purpose.
Shared stories	The digitalised age of information is characterised by an algorithmically-driven “tsunami of information” that can entrench us into present suffering and erode our ability to conceptualise our lives and identities as stories in which we act, interact with other people, and overcome individual and shared problems.	Problematic, pathologising narratives with an exclusive focus on treating disorders tend to dominate youth mental health. By shifting the focus to strengthening life skills and identity, health-promoting initiatives can help youths to develop healthy agency to overcome everyday stressors and shortcomings as they learn to navigate society and the healthcare system.

In an era of digital overreliance and social media pressure, in-person group creative activities with social purpose could be an effective way to promote youth health and strengthen much-needed community bonds.

## Contributors

Both authors contributed equally to all aspects of the article.

## Declaration of interests

The authors declare that they have no conflicts of interest. TD declares honoraria as an associate editor for the BMJ Group.
